# A Latency and Coverage Optimized Data Collection Scheme for Smart Cities Based on Vehicular Ad-Hoc Networks

**DOI:** 10.3390/s17040888

**Published:** 2017-04-18

**Authors:** Yixuan Xu, Xi Chen, Anfeng Liu, Chunhua Hu

**Affiliations:** 1 School of Information Science and Engineering, Central South University, Changsha 410083, China; yixuan_xu@csu.edu.cn (Y.X.); xchen@csu.edu.cn (X.C.); afengliu@mail.csu.edu.cn (A.L.); 2 Key Laboratory of Hunan Province for Mobile Business Intelligence, Hunan University of Commerce, Changsha 410205, China; 3 Mobile E-Business Collaborative Innovation Center of Hunan Province, Hunan University of Commerce, Changsha 410205, China

**Keywords:** smart city, data collection, VANET, opportunistic routing, data mining

## Abstract

Using mobile vehicles as “data mules” to collect data generated by a huge number of sensing devices that are widely spread across smart city is considered to be an economical and effective way of obtaining data about smart cities. However, currently most research focuses on the feasibility of the proposed methods instead of their final performance. In this paper, a latency and coverage optimized data collection (LCODC) scheme is proposed to collect data on smart cities through opportunistic routing. Compared with other schemes, the efficiency of data collection is improved since the data flow in LCODC scheme consists of not only vehicle to device transmission (V2D), but also vehicle to vehicle transmission (V2V). Besides, through data mining on patterns hidden in the smart city, waste and redundancy in the utilization of public resources are mitigated, leading to the easy implementation of our scheme. In detail, no extra supporting device is needed in the LCODC scheme to facilitate data transmission. A large-scale and real-world dataset on Beijing is used to evaluate the LCODC scheme. Results indicate that with very limited costs, the LCODC scheme enables the average latency to decrease from several hours to around 12 min with respect to schemes where V2V transmission is disabled while the coverage rate is able to reach over 30%.

## 1. Introduction

The Internet of Things (IoT) envisions a promising future for the traditional Internet industry and society [1,2,3]. Full intelligentialization can be considered as the ultimate goal of the Internet of Things [1,2]. However, one key issue related to achieving such intelligentialization is how to leverage the ubiquity of sensor-equipped devices to collect data at low cost and on a large scale [1,4,5]. Further organization, integration, and management of collected big data [6,7] enable the construction of knowledge-based decision systems, providing a new intelligent paradigm for solving complex applications with urgent demands, such as surveillance systems, remote patient care systems, intelligent traffic management, and automated vehicles in intelligent transportation systems [1,8,9].

The smart city is one precise realization of intelligentialization in the Internet of Things, which is able to monitor and sense various kinds of objects through a large number of low-cost sensing devices deployed in the city [10,11]. These sensing devices (e.g., wireless sensor nodes) tend to have simple structures, limited energy, and small transmission distances [12,13], on the other hand, some security issues [14,15,16,17,18] still exist among them—being easily attacked by clone attack, DdoS [15]. Nevertheless, due to the lower price and the convenience of deployment [19,20,21,22], they are still widely used in many applications for data collection and environment surveillance [4,10,23,24,25]. After the process of collecting data from devices, the smart city will refine and process the data, and finally make smart decisions to improve the overall quality of the smart city [1,7]. Due to the rich kinds of sensing devices and communication protocols, people have great confidence in realizing smart cities. However, one key premise for this realization is successfully obtaining the data sensed by devices [2,10]. In other words, the so-called intelligence in the smart city depends heavily on the collected data.

With the continuous developments in sensing devices, we have witnessed a great increase in the number of methods related to data collection [4,26]. For example, some sensing devices are connected to decision-making institutes in smart cities (called data centers below) through wired networks. The reliability and high-speed of wired networks enable real-time monitor and data collection from sensing devices. However, many problems faced by a smart city cannot be easily solved through using sensing devices with wired connections. In many situations, the monitored or sensed objects are temporary or located at remote areas without infrastructures that allow wired transmission. Building specialized infrastructures for monitoring and sensing these objects can be extremely costly [4,10]. Taking the development of a city as an example, the construction and reconstruction of the fundamental infrastructures in the city (e.g., streetlights, garbage cans) tend to face the problem above. In order to sense the states of these infrastructures, sensing devices are usually embedded into them. For instance, through embedding sensing devices into garbage cans, people are able to trace their trash levels and report their states to a data center; through sensing devices deployed on streetlights, people are able to know whether they are damaged [4]. In conclusion, deploying sensing devices makes it possible for the smart city to effectively monitor and manage a large number of fundamental infrastructures, providing a better environment to people. However, apart from the great number of these infrastructures, they are also widely distributed in the smart city and deployed dynamically with the development of city. Therefore, building wired connections for sensing devices deployed on such infrastructures can be costly and hard to maintain in the future.

Another kind of method related to data collection in a smart city is using wireless connections, for which one feasible solution is equipping each sensing device with a SIM card. With the help of SIM cards, sensing devices are able to directly upload sensed data to data centers. However, such solutions also have disadvantages. Generally, the cost of one SIM card is about a few dollars, almost equivalent to the cost of one sensing device. More importantly, apart from the costs of the SIM cards, transmission service fees also need to be taken into consideration. Therefore, investments in SIM cards and their subsequent data transmission can easily exceed that in the sensing devices themselves. Besides, wireless transmission with SIM cards consumes more energy, which reduces the lifetime of the sensing devices.

In conclusion, obtaining real-time data in a smart city requires large investments, regardless of whether sensing devices with wired connections or wireless connections are used. However, a great proportion of the data in a smart city has the characteristic of being latency-tolerant. Take sensing devices used to monitor green plants in the city as an example, there is no need for the data related to soil moisture to be transmitted to data centers instantly. Briefly speaking, the time spent on transmitting latency-tolerant data may be longer than one day in a smart city, but such latency is still considered to be acceptable. Besides, some kinds of data on smart city is loss-tolerant. For example, with air pollution sensors deployed on mobile vehicles, these vehicles are able to collect data on air pollution (e.g., PM_2.5_) when moving around the city [27]. Air pollution data then can be obtained by data centers through near field wireless communications when mobile vehicles pass by data centers. Due to the fact that data on air pollution related to one specific area will be collected by all vehicles that enter this area, the possibility that these data can be obtained by smart city will still increase even if there exists no reliable connection between mobile vehicles and data centers, as many copies on these data exist among the mobile vehicles (reliable connection means that schemes on retransmitting are adopted when data loss happens). Even in the worst case that no data related to this area is available, people are still able to make an estimation provided that data on neighbor areas has already been obtained by data centers.

In consideration of the characteristics of latency-tolerance and loss-tolerance, transmitting this kind of collected data through real-time methods will represent a great waste of public resources. One effective and economical solution that adapts to these data is using mobile vehicles as “data mules” [16]. Broadly speaking, mobile vehicles serve as carriers of collected data. When these mobile vehicles pass by sensing devices, the collected data will be transmitted to and stored in them. On the other hand, data centers are also able to receive collected data when mobile vehicles pass by the data center. Through analyzing real-world trajectories of taxis, Bonola et al. demonstrated the feasibility on this kind of solution [10], which will be further explained in the following section.

Although there have already been researches on using mobile vehicles as “data mules” to collect data, several key issues deserve further study: (1) in previous studies, collected data will only be transmitted to data centers when mobile vehicles pass by data centers. However, in a large-scale smart city, a great number of mobile vehicles tend not to pass by data centers within a relatively short time. As to these vehicles, data collected by them cannot be fully utilized. Therefore, one important problem is how to obtain data collected by those mobile vehicles without passing by data centers; (2) as far as we know, the latency of collected data was rarely considered in previous studies. The latency is defined as difference between one data packet’s collection time and its time of being received by data centers. Intuitively, a smart city is able to respond to incidents faster with a relatively smaller latency, leading to improvements on the overall quality of service. However, how to reduce the latency of collected data in smart cities is rarely covered by previous studies; (3) the problem of the coverage of collected data is also neglected by previous studies. Assume that using mobile vehicles as “data mules” is able to collect data on k different areas while the total number of areas in smart city is n. Coverage of collected data is defined as the ratio of k to n. Applications related to environment monitoring in smart city (e.g., PM_2.5_, noise) are associated with coverage directly. With a large coverage, detailed distributions on these data can be obtained, along with better decisions on environment protection.

Based on the analysis above, a latency and coverage optimized data collection (LCODC) scheme is proposed in this paper to effectively collect data on smart city with opportunistic communication style. The main contributions of the LCODC scheme include:

- A LCODC scheme is established to collect data from sensing devices through “data mules” (taxis or other vehicles). Apart from transmission between mobile vehicles and data center (V2D), the LCODC scheme also enables mobile vehicles to exchange data with each other. Compared with previous schemes with V2D transmission alone, vehicle to vehicle transmission (V2V) further mitigates the problem of the deficiency of collected data and increases the coverage rate.
- We propose two important performance metrics (latency and coverage) to evaluate the performance of large-scale opportunistic data collection, along with a series of optimization algorithms to improve the performance on collecting data. Briefly speaking, the LCODC scheme converts the initial problem into dual-optimization under constrained situations: (a) in the LCODC scheme, the internal memory of mobile vehicles is considered to be limited. When there is no space for newly collected data, mobile vehicles need to discard data with less importance. Therefore, the LCODC scheme converts the trade-off between data into an optimization problem under the reality of limited internal memory size of mobile vehicles; (b) when mobile vehicles meet each other, they need to decide whether to transmit data to the neighboring vehicle will improve the overall data collection performance or not. The LCODC scheme converts this problem into an optimization problem of calculating the priority for each vehicle to achieve a better performance.
- Compared with previous studies, a large-scale and real-world dataset is used to evaluate the LCODC scheme. Our comprehensive simulation demonstrates that the LCODC scheme can reduce the average latency of data from several hours to 12 min, and the coverage rate of the whole city is able to reach over 30%.

The remainder of this paper is organized as follows: in Section 2, related works are reviewed. The system model and problem statement are described in Section 3. In Section 4, details on the LCODC scheme are presented. In Section 5, experimental results, comparisons, and impacts of parameters are discussed. We finally conclude the paper in Section 6.

## 2. Related Work

In this part, we give a brief overview on proposed methods that show superiority on collecting data on smart cities [10,28,29]. Specifically, Long-Range, Wide-Area Network (LoRaWAN), Wi-SUN, Airborne Sensors, and Data Mules are introduced.

### 2.1. LoRaWAN

LoRaWAN is a kind of wireless communication network that has characteristics of low power consumption, large coverage rate, and low data rate. In LoRaWAN, fixed gateways in the network are used to forward data from sensing devices to data centers. Technical reports have claimed that with gateways deployed in the network, LoRaWAN can easily cover hundreds of square kilometers. Besides, due to low data rate, battery lifetime of sensing devices can reach multi-year [29]. One important contributor of the long range of LoRaWAN is its use of the sub-GHz ISM (Industrial Scientific Medical) band, which has inherent advantages for coverage and penetration compared with other bands like 2.4 GHz and 5 GHz.

Although with alluring benefits, one problem occurs when building LoRaWANs: the sub-GHz ISM band is available only in a third of the world, and many countries don’t have any band available [30]. Therefore, it can be nearly impossible to extensively adopt technologies like LoRaWAN. Besides, problems still remain in countries with the sub-GHz ISM band in that apart from LoRaWAN, many technologies or companies also occupy this good-quality and free band, leading to even more serious transmission collisions and degraded performance of the LoRaWAN.

### 2.2. Wi-SUN

Like LoRaWAN, the Wireless Smart Utility Network (Wi-SUN) is another kind of wide area network based on IEEE 802.15.4 g. Apart from the sub-GHz ISM band, Wi-SUN also supports the 2.4 GHz ISM band, which greatly increases its practicability. However, this also leads to a smaller range (5–10 km) than the aforementioned LoRaWAN (up to 100 km) [31]. To expand its coverage, Wi-SUN enables sensing devices to communicate with each other. For example, when one sensing device is out of the range of gateways, it is still able to upload data with the help of relay devices between itself and gateways [31]. Besides, the participation of relay devices also increases the flexibility of Wi-SUN.

However, this process also increases the energy consumption of the relay devices, leading to a shorter lifetime of these devices. To make it worse, some relay devices may have to forward data collected by a large number of other sensing devices considering the complex topology, which will aggravate the unbalance energy distribution of sensing devices. Furthermore, once these relay devices run out of energy, other sensing devices will be unable to upload data even if they can still collect it [32]. This phenomenon leads to a decrease on the efficiency of data collection. Although with existing methods, this problem can be mitigated, the complexity of communication also increases [33,34].

### 2.3. Airborne Sensors

Using airborne sensors to collect remote data is another viable method. Information on remote areas can be extracted from optical images or Light Detection and Ranging (LIDAR) data sensed by airborne sensors [35]. Compared with LoRaWAN, one advantage of using airborne sensors is that it requires no wireless communication between sensing devices and data centers. Therefore, remote data collection costs can be greatly reduced. However, this also limits the number of data types that can be obtained. For example, data on streetlights is much harder to collect than PM_2.5_ data with this method. Besides, weather conditions will severely disrupt its ability to collect data and it is time-consuming and challenging to extract information from optical images and LIDAR data. These disadvantages reduce the practicality of using airborne sensors to collect remote data on smart cities.

### 2.4. Data Mules

One architecture used to collect data in sensor network was proposed in [28], where mobile entities in the environment will collect data, store it, and finally drop off the data at fixed locations, which are thus vividly called “data mules”. Through reasonable controls on data mules, the architecture is able to effectively collect data from sensors deployed over a large geographical area. Using data mules to collect data has advantages of low power consumption and low cost on building infrastructures. However, one important disadvantage of this architecture is high latency, which remains undiscussed in [28] due to lack of space.

Bonola et al. extended the concept of data mules in [10], leading to a more realistic mobile entity called oblivious data mules. Compared with original data mules, oblivious data mules have no controls, which conforms better to real-world mobile entities. Taking taxis as an example, which can be considered a typical kind of oblivious data mules, they move independently in the city and according to customers’ needs. In other words, their movements have the characteristics of being random and not following planned paths. Apart from the advantage of being more real, the collected data coverage rate can also increase with oblivious data mules. In [10], Bonola et al. used a real-world dataset on Rome to illustrate that even with a very limited number of oblivious data mules, the coverage of downtown areas in the city was able to reach over 80% within a day. However, the coverage of remote areas, which is of great importance in many applications, was not taken into consideration in [10]. Intuitively, the number of times that data mules enter remote areas is much smaller than that of downtown areas, leading to a lower coverage of remote areas. Besides, time spent on entering remote areas and returning back into downtown areas can be very long, which aggravates the problem on high latency with this method.

Compared with the aforementioned three methods, using data mules to collect remote data is free from long range wireless transmission problems. In fact, this method requires only near field communications between sensing devices and data mules, and between data mules and data centers. Besides, these mobile data mules are easier to manage and maintain compared with fixed gateways deployed in the network. In addition, the number of data types and data availability are not constrained like when using airborne sensors.

## 3. System Model and Problem Statement

### 3.1. System Model

In this section, we first introduce three important components of the LCODC scheme: (1) mobile vehicles; (2) data packets; (3) data centers, along with the key parameters used to define them. After then, a usage scenario is presented, based on which the experiment is conducted.

#### 3.1.1. Mobile Vehicles

Mobile vehicles indicate mobile entities in the smart city (e.g., taxi), along with the sensors deployed on them. When entering a new area, sensors deployed on mobile vehicles will autonomously collect data of interest related to this area. These data will then be encapsulated into data packets and stored in the internal memory of the mobile vehicles. Apart from data collection and storage capacities, mobile vehicles also have the ability to communicate with neighboring vehicles. To effectively distinguish differences between mobile vehicles, priorities used to measure the reliability are introduced in LCODC scheme. The definition of reliability will be further explained in Section 4.5. Assume that the total number of mobile vehicles in the smart city is M, then they can be represented by two different sets $\left\{S_{1},S_{2},\ldots ,S_{M}\right\}$ and $\left\{P_{1},P_{2},\ldots ,P_{M}\right\}$, where S denotes the unique IDs of mobile vehicles while P denotes their priorities. Since there is a one-to-one correspondence between mobile vehicles and sensors, they are represented by mobile vehicles alone in the following subsections, under the premise of causing no ambiguity.

#### 3.1.2. Data Packets

Due to the limited internal memory size of mobile vehicles, the number of data packets that can be stored in one mobile vehicle has an upper limit. When there is no space for newly generated data packets, mobile vehicles will discard packets of less importance in the LCODC scheme. Generally, a weight is calculated for each data packet stored in internal memory and the newly generated data packets, and mobile vehicles will discard data packets with relatively smaller weights. Assume that the upper limit is N, then the data packets stored in mobile vehicle i can be represented by $\left\{D_{1}^{\left(i\right)},D_{2}^{\left(i\right)},\ldots ,D_{N}^{\left(i\right)}\right\}$ and $\left\{W_{1}^{\left(i\right)},W_{2}^{\left(i\right)},\ldots ,W_{N}^{\left(i\right)}\right\}$, where $D_{j}^{\left(i\right)}$ is the unique packet ID of the j-th data packet stored in mobile vehicle i, and $W_{j}^{\left(i\right)}$ is the weight on data packet $D_{j}^{\left(i\right)}$.

#### 3.1.3. Data Centers

Data centers are considered to be fixed devices in the smart city, and also the destinations of the transmitted data packets in the LCODC scheme. Data will be refined by data centers for real applications. For example, in VTrack [36], data centers are able to provide omnipresent traffic information and NoiseTube [37] can make noise maps if enough data is available. Generally, in order to increase the possibilities of communication between mobile vehicles and data centers, they should be deployed in areas where mobiles vehicles pass by most frequently. Such areas are called urban areas in this paper. Assume that there are a total number of K data centers being deployed in a smart city, then they can be denoted as one set $\left\{C_{1},C_{2},\ldots ,C_{K}\right\}$.

#### 3.1.4. Application of the LCODC Scheme

**Figure 1:**
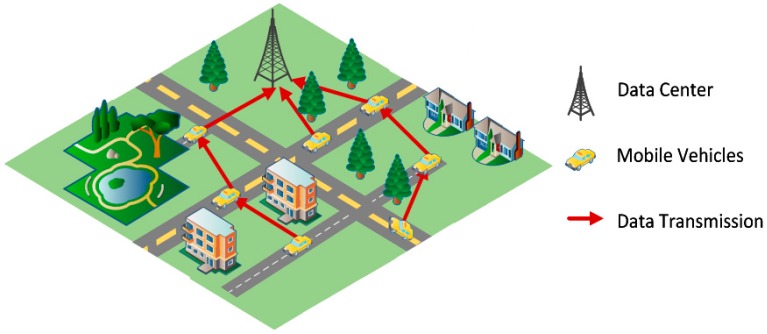
One application of the LCODC scheme.

The quintuple (S,P,D,W,C) consists of all elements used to illustrate and implement the LCODC scheme. One usage scenario is presented in Figure 1. Moving taxis in smart city are considered to be mobile vehicles with sensors deployed on them (S). After collecting data packets (D), mobile vehicles will transmit these packets to neighbor vehicles or data centers (C). Upon successfully receiving data packets, data centers will refine them for real applications. Note that apart from interested data on areas (e.g., PM_2.5_, noise, and traffic), key information on collected data is also integrated into data packets, such as collection time and location.

### 3.2. Problem Statement

Nowadays, much real-time information is available in smart cities. For example, people can request information on PM_2.5_ and NoiseTube through browsing government sites. Real-time traffic flow information is also available through mobile map applications. However, owing to the large costs of constructing and maintaining these sensing devices, currently most of them tend to be deployed in urban areas, such as business districts and main streets. This uneven distribution causes real-time information on remotes areas to be inaccurate or unavailable.

Using mobile vehicles in smart city to obtain information on remote areas is an economic and effective data collection pattern. Taking taxis as an example, when taxis enter remote areas, sensors deployed on them can autonomously collect data on these remote areas. After returning back into urban areas, taxis can transmit the collected data to data centers. Such kind of collaboration enables data centers to obtain data about remote areas without building additional infrastructure. In other words, the collected data coverage is improved.

In conclusion, the goal of the LCODC scheme is to obtain real-time data on remote areas through the movement and transmissions of mobile vehicles, under the premise that the number of data centers in the smart city is limited. Below are some formal definitions on the goal of the LCODC scheme:

- Assuming that the smart city can be divided into $N_{a}$ areas, implementing the LCODC scheme enables data centers to obtain data from $N_{m}$ different areas. The first goal is to maximize the coverage rate $N_{m}/N_{a}$ of the LCODC scheme.
- Assuming that the collection time of data packet i is ${t_{s}}^{\left(i\right)}$, and it is received by a data center at time ${t_{e}}^{\left(i\right)}$. The second goal of the LCODC scheme is to minimize the latency of data packets i, which is the difference between ${t_{e}}^{\left(i\right)}$ and ${t_{s}}^{\left(i\right)}$:
  (1) $$l_{i}={t_{s}}^{\left(i\right)}-{t_{e}}^{\left(i\right)},\;{t_{e}}^{\left(i\right)}\geq {t_{s}}^{\left(i\right)}.$$

Therefore, the average latency of data packets in smart cities $L_{average}$ can be obtained according to Equation (2), where D is the total number of data packets received by data centers:

(2) $$L_{average}=\left(\overset{D}{\underset{i=1}{\sum }}l_{i}\right)/D.$$

Equation (3) gives a summary of aforementioned goals:

(3) $$\{\begin{array}{c} \max\;N_{m}/N_{a}\\ \min L_{average} \end{array},$$

We then present several problems to be solved before achieving the goal of the LCODC scheme.

● The problem of measuring the importance of different data packets. 

Intuitively, data packets are generated at different locations and times. According to the aforementioned goals, packets from remotes areas and with low latency should have higher weights compared with other packets. Therefore, one problem in the LCODC scheme is how to correctly measure the importance of data packets.

● The problem of making sure data packets with higher weights have the priority of being transmitted to data centers.

After correctly measuring the importance of different data packets, an effective scheme for transmission between mobile vehicles is needed to make sure that important packets can be transmitted to data centers as soon as possible. The typical problem depicted in Figure 1 is described as follows: 

After collecting data on remote areas, a mobile vehicle remains in the remote areas. At the same time, another vehicle approaches and intends to return to the urban areas. Transmission between two vehicles should enable the data stored in the first vehicle to have a higher possibility of being transmitted to and stored by the second vehicle. Therefore, in order to solve this problem, an important part of the LCODC scheme is designing vehicle to vehicle (V2V) transmission.

● The problem of avoiding aggravating broadcast storm problems in urban areas.

One common problem on V2V transmission is known as the broadcast storm problem [38]. Specifically, there tend to exist many mobile vehicles in an urban area at the same time. Simultaneous transmission between them will cause severe channel collisions, leading to a decrease of channel utilization. Besides, owing to the highly dynamic topology formed by mobile vehicles, it is very likely that receivers have already left the transmission range of senders after a previous transmission failure. Therefore, in order to improve the latency and coverage performance, any designed scheme should have solutions to mitigate the broadcast storm problem.

## 4. LCODC Scheme

### 4.1. Overview

The LCODC scheme is a kind of data collection scheme in vehicular ad hoc networks that is specialized for optimizing the coverage and latency of collected data. One important feature of the LCODC scheme is that collected data in a smart city is considered to be latency-tolerant and loss-tolerant, which enables the broadcast storm problem to be further mitigated. On the other hand, data related to instantaneity (e.g., information about car accidents) may not perform well if transmitted through the LCODC scheme.

Compared with other proposed schemes, the LCODC scheme has the advantages of requiring no extra supporting devices and information related to smart city, along with its simplicity and easy implementation. For example, broadcast devices located at intersections are needed in several schemes to facilitate data transmission, which is costly to build and difficult to maintain. Besides, some schemes rely heavily on information related to roads, such as average speed and traffic flow [39]. Considering the large scale of smart city, such information on remote areas can be hard to obtain. In addition, average speed and traffic flow cannot be simply viewed as constant parameters in a smart city. Intuitively, great differences exist between the traffic flow at rush hour and midnight. The time-dependency greatly escalates the complexity of schemes based on such information and influences their final performance.

The information needed by the LCODC scheme is only the GPS trajectories of mobile vehicles in smart cities. With enough data on trajectories, the LCODC scheme is able to correctly find out the distributions in urban areas and remote areas using big data analysis approaches, along with patterns related to the movements of mobile vehicles. These information serves as the foundation of the LCODC scheme. With one comprehensive study on the patterns in the smart city, waste and redundancy in the utilization of public resources can be reduced, leading to a simpler and more economical data collection scheme.

In general, the LCODC scheme consists of three primary sub-schemes: (1) scheme for deciding the location of data centers; (2) scheme for vehicle to vehicle (V2V) transmission; (3) scheme for vehicle to device (V2D) transmission, which will be illustrated in details in the following sections.

### 4.2. Running States of Mobile Vehicles

Before formally presenting the design details of the LCODC scheme, an explanation of the running states of mobile vehicles is provided to facilitate further discussions. In the LCODC scheme, the running states of mobile vehicles can be divided into four different kinds: (1) collection state; (2) sensing state; (3) dumping state; (4) transmitting state. Switching between these states enables the collected data to be exchanged between mobile vehicles or transmitted to data centers, and finally achieves the goal of obtaining recent data about remote areas. Below are detailed explanations of the four different states:

● Collection state

When mobile vehicles enter a new area, the sensors deployed on them will autonomously switch into collection state and collect data of interest about this area. These data will then be stored in the internal memory of the mobile vehicles.

● Sensing state

The process of sensing neighboring mobile vehicles and receiving data packets is called the sensing state. Specifically, periodic beacon messages are sent by mobile vehicles in the sensing state. As a result, every vehicle is able to detect neighboring vehicles according to these beacon messages. Apart from receiving beacon messages, mobile vehicles also receive data packets transmitted by neighboring vehicles when in this state.

● Dumping state

Mobile vehicles will switch into dumping state when passing by data centers. In this state, mobile vehicles will attempt to transmit the data stored in their internal memory to the data centers. Since data centers are considered to be the data destinations, there is no need for these data to be further transmitted between mobile vehicles. Therefore, mobile vehicles will free up internal memory after finishing transmitting data to data centers. This process is vividly named the dumping state.

● Transmitting state

**Figure 2:**
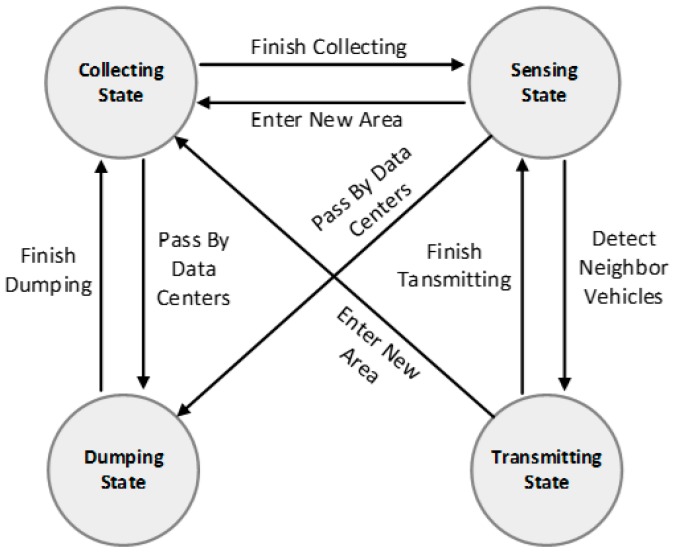
State transition diagram of the running states of mobile vehicles.

Upon detecting neighboring vehicles, mobile vehicles will autonomously switch into transmitting state. When in this state, it is possible for vehicles to exchange data with neighboring vehicles. However, the broadcast storm problem will aggravate acutely when these vehicles are located in urban areas. Therefore, the sub-scheme for transmission between mobile vehicles should take actions to mitigate the broadcast storm problem. A brief state transition diagram of the running states of mobile vehicles is shown in Figure 2.

### 4.3. Deciding the Location of Data Centers

The first sub-scheme is used to find the locations of data centers through data mining on historical GPS trajectories of mobile vehicles. With historical GPS trajectories, distributions in urban areas and remote areas in the smart city can be found. Building data centers in those urban areas can effectively increase the frequency of transmission between mobile vehicles and data centers, enabling more collected data to be received by the data centers. However, choosing areas with mobile vehicles passing by most frequently ignores the coverage rate performance.

Real-world information on the trajectories of mobile vehicles is usually represented by a series of trace points in the map. Therefore, the first sub-scheme can be converted into a clustering problem that aims to find out the internal structure of trace points [40]. Clustering will divide these trace points into different classes, with a high similarity between points in the same class and a low similarity between points in different classes. In the first sub-scheme of the LCODC scheme, Euclidean distance is adopted to measure similarities while different classes are represented by data centers. In conclusion, the LCODC scheme converts the sub-scheme for deciding the location of data centers into a clustering problem.

For convenience, the LCODC scheme will first divide the smart city into square areas of the same size. Compared with other methods, such a partition requires no information about the layout of the smart city, and thus is much easier to implement. In the following sections, the word “area” is replaced by “grid” to reflect the partition method adopted by the LCODC scheme.

In the LCODC scheme, location information about different grids alone is used to decide the locations of data centers. Assume that the historical GPS trajectories cover a total number of M grids in the smart city, with each grid represented by its center’s longitude and latitude, then the location information of these grids can be expressed as a matrix with size M×2. Each row in the matrix corresponds to one specific grid while the first and second column separately denote information about longitude and latitude.

Clustering will divide the grids into K different classes, with each of these grids $x_{i}$ belonging to the class that corresponds to the nearest data center. The goal of this sub-scheme is to decide the locations of data centers that attempt to minimize square error E:

(4) $$E=\overset{K}{\underset{i=1}{\sum }}\underset{x\epsilon D_{i}}{\sum }{\left({∥x-D_{i}∥}_{2}\right)}^{2},$$

where ${∥*∥}_{2}$ is L2-norm, x in the parenthesis indicates grids whose nearest data center is $D_{i}$, and the final location of data center $D_{i}$ can be obtained through averaging locations of x. The value of E can be viewed as a measurement on the similarity among grids that belong to the same data center.

In conclusion, clustering algorithms are adopted in this sub-scheme to decide the locations of data centers. Before clustering, location information alone is used to construct the feature vector for each grid covered by historical GPS trajectories. The final locations of data centers can be obtained through averaging the locations of grids in the corresponding class. Section 5.3.1 shows clustering results on a real-world, large dataset with different clustering algorithms.

### 4.4. Vehicle to Device Transmission

In vehicle to device transmission (V2D), device refers to data centers deployed in the smart city. V2D transmission happens when mobile vehicles pass by data centers. Therefore, this sub-scheme denotes actions taken by mobile vehicles in the dumping state. With the help of the widely used global positioning system, we assume that mobile vehicles in the smart city can easily obtain information about their current locations. Therefore, when passing by data centers, they will autonomously switch into the dumping state shown in Figure 2. Besides, we assume that vehicle to device transmission is instantaneous, which can be achieved through technologies like Ultra-Wideband (UWB). Researchers have reported that with UWB, the measured peak transmission speed can reach over 50 Mbps within a short range [41]. When passing by data centers, the distance between mobile vehicles and data centers can be regarded as relatively short. Therefore, mobile vehicles are able to finish transmitting data within several time slots with UWB. To simplify the model, we make this assumption and focus on transmission between mobile vehicles.

Due to the existence of vehicle to vehicle transmission, there tends to be many copies of a specific data packet among mobile vehicles. Therefore, the possibility that a specific data packet can be received by data centers will be improved through transmitting copies by different mobile vehicles. To avoid aggravating the broadcast storm problem in grids where data centers are located, mechanisms related to Quality of Service (QoS), such as sending ACKs (ACKnowledgements) as a feedback, are not adopted during V2D transmission.

Since the range of wireless transmission is very limited, it can be difficult for vehicles to detect potential collisions because of signal attenuation, leading to the so called hidden terminal problem. However, in our model, V2D transmission happens when mobile vehicles and the data center are located in the same grid. With a reasonable grid size, the hidden terminal problem can be mitigated. Therefore, we adopt P-Persistent Carrier Sense Multiple Access with Collision Detection (P-Persistent CSMA/CD) protocol in V2D transmission to avoid collisions when all mobile vehicles attempt to transmit data to the data center, which is briefly expressed as below:

When a mobile vehicle finishes preparing data to be transmitted, it will first intercepts the channel. If the channel is idle, data will be transmitted with the probability p or is pushed off to the next time slot with the probability 1−p. The situation on the next time slot is similar to the current time slot. When transmitting, it will keep detecting potential collisions. If they exist, the transmission process will be terminated immediately and restarted after a random time interval.

In conclusion, when passing by data centers, mobile vehicles will switch into the dumping state and start transmitting the data stored in their internal memory according to the P-Persistent CSMA/CD protocol. After transmission, the mobile vehicles will clear their internal memory and switch back into a collecting state. Algorithm 1 shows the pseudo-code of V2D transmission from the perspective of mobile vehicles:
**Algorithm 1:** Vehicle to Device Transmission (V2D)1: **While** true2:   **If** passing by data centers3:    switch into dumping state;4:    start transmitting data to data centers;5:    **If** finishing transmitting6:     clear internal memory;7:    **Else**8:     attempt to transmit at next time slot;9:     go to step 5;10:   **End**11:   switch into collecting state;12:  **End**13: **End**

### 4.5. Vehicle to Vehicle Transmission

Compared with the aforementioned two sub-schemes, vehicle to vehicle transmission (V2V) can be considered as the core of the LCODC scheme. On the one hand, the participation of V2V transmission greatly facilitates data flow in the smart city compared with other schemes equipped with V2D transmission alone. On the other hand, mobile vehicles will discard data with less importance according to this sub-scheme when there is no memory space for newly collected data, which directly influences the final performance of LCODC scheme. Before formally describing the sub-scheme for V2V transmission, we first present several assumptions made in the LCODC scheme:

- Each mobile vehicle has access to information on its current location through triangulation or the global positioning system (GPS).
- Traffic flow information about each grid in the smart city is pre-stored in mobile vehicles. Therefore, mobile vehicles are able to easily obtain such information and encapsulate it into the header of data packets.
- Neighboring vehicles can be detected by mobile vehicles in sensing state through receiving beacon messages broadcast by them, along with key information about neighboring vehicles (e.g., ID, weight), which is integrated into beacon messages.

Considering the current widely used navigation systems of mobile vehicles and developments in equipped vehicle devices (e.g., microcomputers integrated into mobile vehicles), we assume that the assumptions above are reasonable and feasible. Besides, mobile vehicles can then easily locate their corresponding grid using location information and pre-stored grid information. Particularly, we assume that the current location of a mobile vehicle is (m,n) while that of the bottom-left corner of the smart city is $\left(m_{0},n_{0}\right)$, then the corresponding grid index of this mobile vehicle along the longitude $Index_{X}$ and latitude $Index_{Y}$ can be calculated as follows:

(5) $$Index_{X}=\left\lceil (m-m_{0}\right)/g_{size}\rceil ,m\geq m_{0}$$

(6) $$Index_{Y}=\left\lceil (n-n_{0}\right)/g_{size}\rceil ,n\geq n_{0}$$

where $g_{size}$ is the side length of grids. Note that $m,n,m_{0},n_{0}$ are not original longitude and latitude information. However, they can be easily obtained through transformation provided that the longitude and latitude information of the mobile vehicles and the corner of the smart city is available.

Intuitively, if one mobile vehicle never has any communication with a data center (known as black holes among mobile vehicles), there is no need for other vehicles to transmit data to it. Therefore, a weight used to reflect the reliability of mobile vehicles is introduced in V2V transmission. Besides, the avoidance of such useless transmission also helps mitigate the broadcast storm problem. 

Assume that the weight on the i-th mobile vehicle is $P_{i}$, its value will increase by one when every time this mobile vehicle communicates with a data center. In addition, the initial value of $P_{i}$ is designated as 1.

When a mobile vehicle $S_{i}$ detects neighboring vehicles, it will autonomously switch into transmitting state according to Figure 2. Besides, $S_{i}$ is also able to obtain information on the ID and weight of neighboring vehicles through beacon messages. After that, $S_{i}$ will compare its own weight $P_{i}$ with the weights of all neighboring vehicles, denoted as the set P. The possibility that $S_{i}$ will broadcast collected data to neighbor vehicles is calculated according to the following equation:

(7) $$\{\begin{array}{c} \frac{\max\left(P\right)}{P_{i}},P_{i}\geq max\left(P\right)\\ 1,P_{i}<max\left(P\right) \end{array},$$

where max(P) denotes the largest weight of the neighboring vehicles. According to Equation (7), if the weight of mobile vehicle $S_{i}$ is much larger than that of the neighboring vehicles, there is a high possibility for $S_{i}$ to reserve its own data and transmit it to data centers individually, leading to a mitigation of the broadcast storm problem. Algorithm 2 is the pseudo-code of transmission between mobile vehicles according to the descriptions above from the perspective of sender $S_{i}$:

**Algorithm 2:** Vehicle to Vehicle Transmission (Sender)1: **While** true2:   **If** detecting neighbor mobile vehicles;3:     switch into exchanging state;4:     find max(P) according to beacon messages;5:     **If**
$P_{i}\geq \max\left(P\right)$
6:       broadcast data with probability $\max\left(P\right)/P_{i}$;7:     **Else**8:       broadcast data with probability 1;9:     **End**10:  **End**11: **End**

Since the neighboring vehicles of $S_{i}$ will also go through the process above, it is possible for more than one vehicle to broadcast their stored data. Therefore, transmission schemes used to avoid collisions are also introduced in V2V transmission.

Apart from sending data, mobile vehicles also need to receive data during V2V transmission. After successfully receiving data packets broadcast by other vehicles, mobile vehicles will store it in their internal memory. However, if there is no free space for the received data packets, schemes will be used to decide which data packet should be discarded.

Specifically, mobile vehicles will calculate weights for the received data packets and data packets stored in their internal memory according to the following equation:

(8) $$W_{j}^{\left(i\right)}=k\times \frac{1}{{B_{j}}^{\left(i\right)}}+\frac{1}{\Delta {T_{j}}^{\left(i\right)}},$$

where $W_{j}^{\left(i\right)}$ is the weight of the jth data packet stored in mobile vehicle $S_{i}$, ${B_{j}}^{\left(i\right)}$ is one parameter related to the grid where this data is collected, and $\Delta {T_{j}}^{\left(i\right)}$ is the difference between the collection time of this data packet and the time that V2V transmission happens. k serves as an adjustment factor used to make sure that ${B_{j}}^{\left(i\right)}$ and $\Delta {T_{j}}^{\left(i\right)}$ have relatively similar impacts on the weight of data packets.

The exact value of ${B_{j}}^{\left(i\right)}$ corresponds to the traffic flow information of grids, which refers to the number of times that mobile vehicles enter a grid within a period of time. Assume that within H hours, the number of times that mobile vehicles enter grid i is N, then the traffic flow information of grid i is N/H per hour. Intuitively, remote areas tend to have a smaller value of ${B_{j}}^{\left(i\right)}$, leading to a greater increase on the weight of data packets, which indicates that in the LCODC scheme, data packets collected from remote areas have a superior chance of being accepted by other vehicles. 

As to the value of $\Delta {T_{j}}^{\left(i\right)}$, Equation (9) is used to obtain its real-time value:

(9) $$\Delta {T_{j}}^{\left(i\right)}=t_{now}-{t_{j}}^{\left(i\right)},$$

where ${t_{j}}^{\left(i\right)}$ is collection time of this data packet while $t_{now}$ is the current time. According to Equation (9), data collected recently tends to have a smaller value of $\Delta {T_{j}}^{\left(i\right)}$ compared with data collected a long time ago, which indicates that in the LCODC scheme, recent data packets have a superior chance of being accepted by other vehicles.

After methods on measuring importance of data packets are presented, the pseudo-code of transmission between mobile vehicles from the perspective of receiver $S_{i}$ is described in Algorithm 3.

**Algorithm 3:** Vehicle to Vehicle Transmission (Receiver)1: **While** true2:   **If** receiving data packets from other mobile vehicles;3:     For each received data packet D;
4:       **If** internal memory still has spare space;5:         store D;6:       **Else**7:         compute weights for all packets according to Equation (8);8:         discard the packet with minimum weight;9:       **End**10:    **End**11:  **End**12: **End**

**Figure 3:**
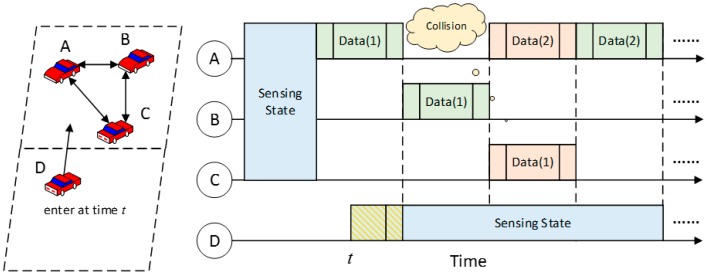
An illustration of vehicle to vehicle (V2V) transmission.

Figure 3 shows data transmission between three different mobile vehicles {A,B,C}. Then, another mobile vehicle D enters the grid at time t. In the beginning, A, B, and C detect each other according to beacon messages. After that, they switch from sensing state into transmitting state and start to exchange data. Detailed information on the following transmission is shown in the figure. At time t, mobile vehicle D enters this grid. Since A, B, and C no longer broadcast beacon messages at time t, D still remains in sensing state but is able to receive data transmitted by other vehicles. Note that at first, it successfully receives part of the first data packet transmitted by A, along with the end tag. D then makes the judgement that this data packet is incomplete and discards it. The subsequent situations on receiving data packets are same for A, B, and C.

### 4.6. QoS Requirements

Although the proposed LCODC scheme focuses on latency-tolerant and loss-tolerant data in smart cities, in this section we demonstrate that Quality of Service (QoS) requirements can also be fulfilled with additional mechanisms. However, this inevitably increases the complexity of the LCODC scheme.

Intuitively, different types of data generated in smart cities have different transmission requirements [9]. For example, congestion and accident information should be transmitted to data centers as soon as possible. Therefore, data packets are classified into classes with different hierarchies in many real-world networks. Assume that a 5-level partition {1,2,3,4,5} is adopted, so data packets with greater value should be transmitted first. Several mechanisms can be added to the LCODC scheme during V2V and V2D transmission to meet this requirement:

(1) With information on the average superiority of data packets stored in one mobile vehicle integrated into its beacon messages, neighboring vehicles are able to know currently which vehicle has the largest average superiority. After that, mobile vehicles in the same grid will transmit data according to the descending order of average superiority.
(2) Different possibilities p can be designated for mobile vehicles with different average superiorities during V2D transmission. Specifically, the mobile vehicle with the larger average superiority is more likely to transmit data in the current time slot.

As to the security, mechanisms on mobile vehicles authentication and data packet evaluation [42,43,44] can be integrated into the LCODC scheme when detecting neighboring vehicles and discarding data packets. Due to lack of space, design details to fulfill QoS requirements is not explored in this paper, and will be covered in our future work.

### 4.7. Summary on LCODC

**Figure 4:**
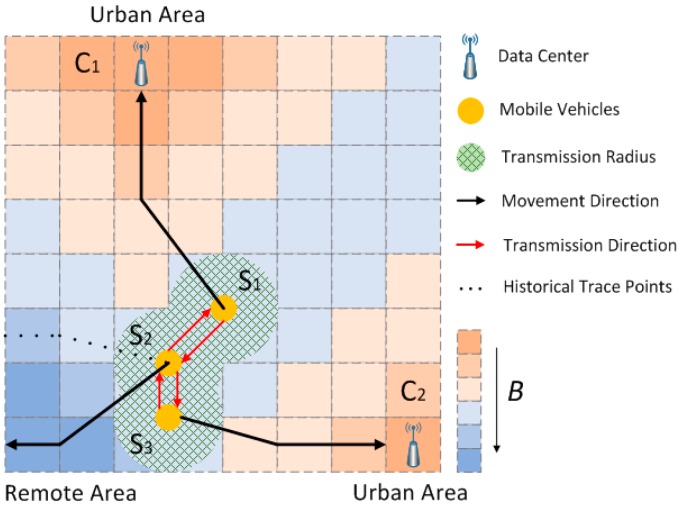
Schematic diagram used to summarize the LCODC scheme.

Figure 4 summarizes the LCODC scheme, with three mobile vehicles ($S_{1},S_{2},S_{3}$) moving toward different grids and two fixed data centers ($C_{1},C_{2}$) located in the grid map.

The locations of two data centers have already been determined according to clustering algorithms and historical GPS trajectories. At present, $S_{2}$ detects two neighboring vehicles through beacon messages sent by $S_{1}$ and $S_{3}$, along with their weight $P_{1},P_{3}$. $S_{2}$ will autonomously switch into transmitting state and compare $P_{1},P_{3}$ with its own weight $P_{2}$. It then uses Equation (7) to calculate the probability of broadcasting data stored in its internal memory. 

The situations of $S_{1}$ and $S_{3}$ are similar to that of $S_{2}$. For simplicity, we assume that $S_{1}$, $S_{2}$, and $S_{3}$ will switch to transmitting state simultaneously while the CSMA/CD protocol is adopted to avoid possible collisions. After broadcasting and receiving data, each mobile vehicle is expected to obtain some data from other devices (due to possible packet loss, small weights of some delivered packets, and decisions according to Equation (7), a mobile vehicle tends not to accept all the packets sent by other vehicles).

Since mobile vehicles keep moving during transmission, the process of broadcasting and receiving can be interrupted unexpectedly. In practice, every data packet should start and end with special tags to inform other devices. If mobile vehicles receive the start tag or end tag alone, they can judge that the transmission is fragmentary.

After obtaining some data from other vehicles, $S_{1}$ and $S_{3}$ will pass by data centers and switch into dumping state. All data stored in their internal memories are transmitted to data centers, which are considered to be destinations. According to the historical trace points of $S_{2}$, its internal memory stores data collected from remote areas. The transmitting state enables these data to have a higher probability of being transmitted to $S_{1}$ and $S_{3}$ since they have larger weights according to Equation (8). Finally, they can be transmitted to data centers faster with the help of $S_{1}$ and $S_{3}$, which achieves the target described in Section 3.2.

## 5. Experiment on the LCODC Scheme

### 5.1. Overview of Experiment

To thoroughly evaluate the latency and coverage performance of the LCODC scheme, the T-Drive dataset is adopted in the experiment, which contains the GPS trajectories of 10,357 taxis from 2 February to 8 February 2008 within Beijing [45,46]. Specifically, trajectories are composed of about 15 million trace points, with each of them containing information on taxi id, collection time, longitude, and latitude. Since T-Drive has the advantage of collecting data from the real world and on a large scale, we assume that using this dataset to prove the superiority of LCODC scheme on latency and coverage rate is persuasive.

**Figure 5:**
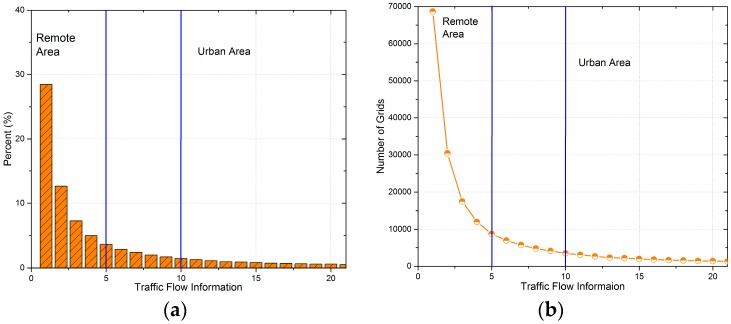
Summary statistics of the T-Drive dataset. (**a**) Percent of grids with different traffic flow information; (**b**) Number of grids with different traffic flow information.

Figure 5 provides summary statistics of the T-Drive dataset. We assume that grids with a number of times that mobile vehicles enter smaller than 6 are remote areas, while grids with a number of times greater than 10 are urban areas. According to Figure 5, around 57% of the grids are remote areas while urban areas account for 33%.

### 5.2. Methodology Introduction

In this section, preprocessing methods on the T-Drive dataset are introduced, along with key parameters used in the experiment. In general, preprocessing methods include gridding of the city and data filtering.

#### 5.2.1. Gridding of the City

The longitude of the smart city ranges from 115.7° E to 117.4° E while the latitude ranges from 39.4° N to 41.6° N, covering an area of over 35,160 square kilometers. Assume that the maximum transmission radius of taxis is r, then the size of square grid is $(\sqrt{2}/2)r\times (\sqrt{2}/2)r$, which ensures that taxis in the same grid are able to detect and communicate with other vehicles. Note that although we ignore possible V2V transmission between mobile vehicles in neighbor grids for convenience in the experiment, the proposed LCODC scheme is not constrained by this preprocessing method.

#### 5.2.2. Data Filtering

**Figure 6:**
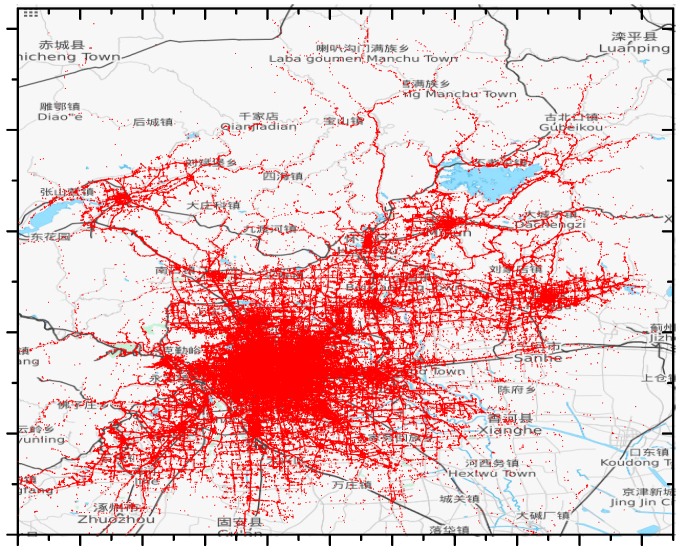
Visualization of the filtered T-Drive dataset (© OpenStreetMap Contributors).

First, points out of city range are discarded. In addition, abnormal points are picked out and filtered, which indicate points that are considered to be too far from their previous points. Consider that time interval between two neighboring points of one taxi is t and maximum speed limit in smart city is v, if the distance between two points is greater than v×t, we assume the latter point to be abnormal. Figure 6 shows the main part of the T-Drive dataset (from 2 February to 8 February) after data filtering (v=80Km/h).

Table 1 presents several key parameters used in the experiment.

### 5.3. Experimental Results

#### 5.3.1. Locations of Data Centers

After pre-processing, we obtain a total number of 241,329 grids with mobiles vehicles passing by, which is greatly smaller than the overall size (2050×3453). Considering the complex terrain of Beijing (mountains, pools, and lots of historic sites), there do exist a large number of grids inaccessible to vehicles.

**Figure 7:**
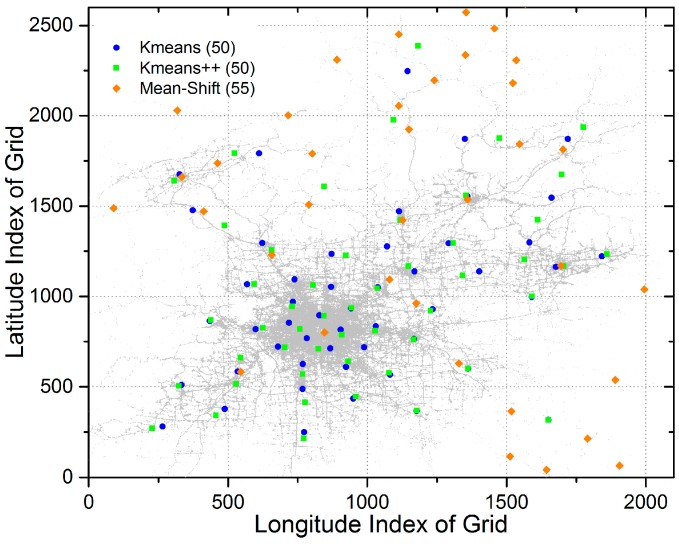
Final locations of data centers with different clustering algorithms.

First, we implement the sub-scheme for deciding the location of data centers. In this part, the adopted clustering algorithms are k-means, k-means++, and mean-shift. In addition, the initial number of data centers K (known as a hyper-parameter) in k-means and k-means++ is set to 50. Figure 7 shows the final locations of data centers with different clustering algorithms. Intuitively, some data centers determined by k-means and k-means++ overlap with each other. Besides, locations of data centers determined by mean-shift have a sparser layout. 

#### 5.3.2. Performance on Latency and Coverage

According to Section 3.2, latency is defined as the difference between the data collection time and the uploading time. Assume that a data packet is generated in time slot i and arrives at a data center in time slot j, then its latency should be $\left(j-i\right)\times t_{interval}$, where $t_{interval}$ is the length of the time interval. In our experiment, $t_{interval}$ is set to 2 min according to Table 1.

Table 2 presents experimental results with three different clustering algorithms, from which we can observe that k-means and k-means++ outperform mean-shift in both average latency and coverage. In the light of the locations of data centers determined by mean-shift in Figure 7, this gap can be reasonable since many data centers are located in remote areas. Also, this phenomenon indicates that locations of data centers have a great impact on the final performance. Therefore, in Section 5.5, we will conduct a comprehensive research on the performance of the LCODC scheme with different numbers and layout of data centers.

**Figure 8:**
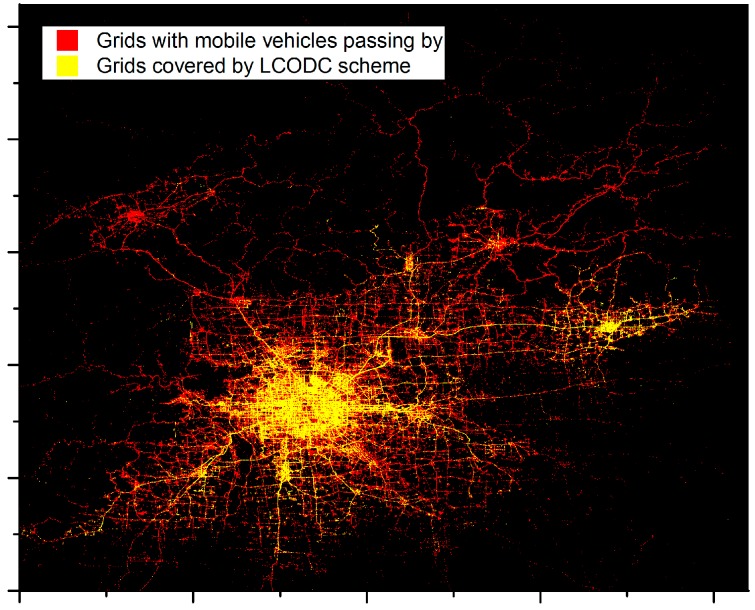
Distribution based on grids covered by uploaded data packets.

**Figure 9:**
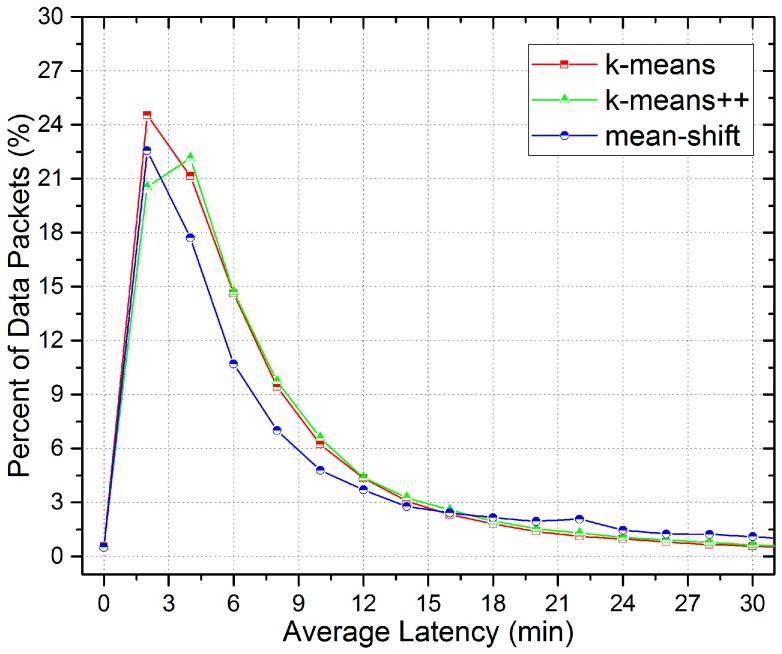
Distribution based on the latency of uploaded data packets.

In addition, the experimental results show the effective transmission between mobile vehicles in the LCODC scheme according to the number of mobile vehicles covered by data packets, which is much larger than the number of mobile vehicles that pass by data centers. In fact, the exact number of mobile vehicles involved in our experiment is 8826 after filtering, while only around half of them have ever passed by data centers. Therefore, without V2V transmission, a great proportion of the data collected by mobile vehicles could never be transmitted to data centers. Figure 8 shows a detailed distribution of the covered grids with k-means++ while Figure 9 presents the distribution based on the latency of uploaded data packets.

### 5.4. Comparison

In this section, we first compare the performance of the LCODC scheme with two fundamental data collection schemes in smart cities. Since a comprehensive comparison with other methods mentioned in Section 2 (e.g., LoRaWAN, Airborne Sensors) can be hard, we will just give a brief illustration of the superiority of LCODC scheme. The two fundamental data collection schemes are described as follows:

● Scheme One

The first scheme discards vehicle to vehicle (V2V) transmission. When entering a grid, each mobile vehicle will autonomously collect data. Upon passing by data centers, these data will be uploaded to data centers.

● Scheme Two

The second scheme is similar to the LCODC scheme, however, instead of using Equation (8) to calculate the weight for each data packet and discarding the data packets with minimum weight, this scheme two discards data packets randomly. The rest is the same as the LCODC scheme.

Table 3 presents the two schemes’ final average latency and coverage performance. From Table 3 we can observe that the LCODC scheme greatly outperforms Scheme One in both latency and coverage, revealing the importance of vehicle to vehicle (V2V) transmission in any data collection scheme, while the LCODC scheme greatly reduces the latency compared with Scheme Two. However, since data collected from remote areas but with large latency will also be discarded by the LCODC scheme, Scheme Two slightly outperforms the LCODC scheme on coverage.

As to the comparison with LoRaWAN, due to the lack of exact parameters, we assume that the coverage radius of gateways is 500 m, which is approximately the coverage radius of a mobile communication base station using the 900 MHz band in urban areas. According to the aforementioned experimental results, the LCODC scheme covers an area of $269{\;\mathrm{km}}^{2}$. To achieve the same coverage, the number of gateways needed can easily reach over 300. Note that we ignore the complex layout of this area. Compared with using airborne sensors, the LCODC scheme shows great superiority in average latency since it can be impractical to obtain data through airborne sensors within such a time interval, let alone the fact that number of data types obtained by airborne sensors are greatly limited.

### 5.5. Analysis of the Experiment

In this part, we conduct a comprehensive analysis on the performance of the LCODC scheme and the impacts of key parameters. First, we dig into the experimental results of the LCODC scheme. Then, the impacts of the number of data centers and their locations are presented. Finally, a study of the impact of the internal memory size of mobile vehicles is conducted. Besides, to obtain more information on the performance of the LCODC scheme, an additional part is included in this section. Specifically, we separately pick out two sub-datasets according to their collection time (10:00 p.m.–6:00 a.m. and 8:00 a.m.–8:00 p.m.) to study the difference in the performance of the LCODC scheme at different times of a day.

#### 5.5.1. Analysis on Experimental Results

**Figure 10:**
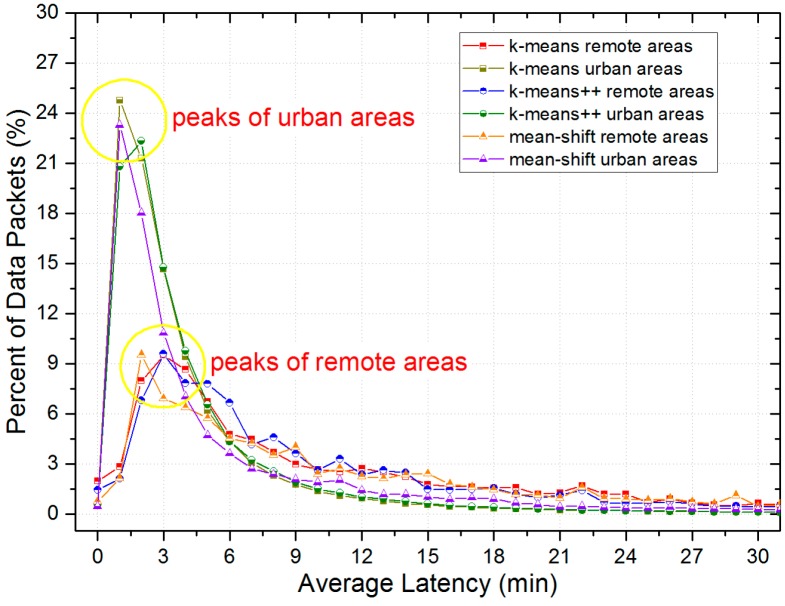
Detailed distribution of the average latency.

**Figure 11:**
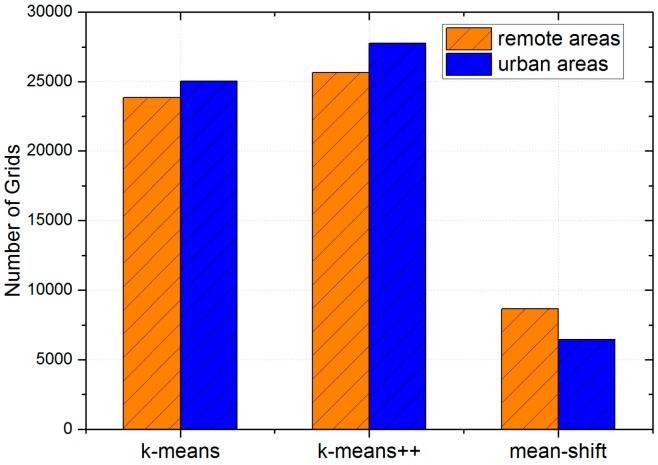
The number of remote and urban areas covered by uploaded data packets.

**Figure 12:**
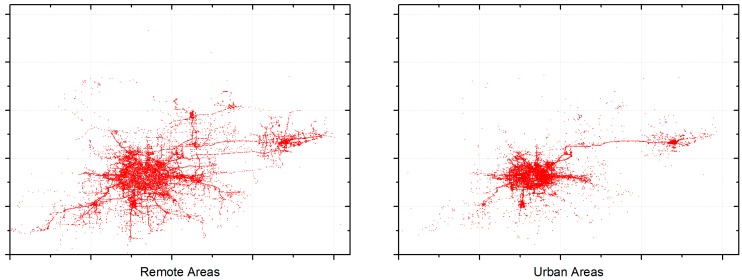
Distributions of remote and urban areas covered by uploaded data packets.

According to Figure 5, we define grids with traffic flow information smaller than 6 as remote areas, while grids with that greater than 10 are defined as urban areas. Therefore, we first find out the exact distribution of uploaded data packets. Figure 10 presents the detailed distribution of the average latency and Figure 11 presents the number of remote and urban areas (grids) covered by uploaded data packets. Besides, Figure 12 shows the distribution of remote and urban areas covered by data packets.

Intuitively, Figure 10 shows that data packets related to urban areas tend to arrive at data centers faster than data packets related to remote areas, as compared with peaks of remote areas, peaks of urban areas have a greater proportion (higher) and a smaller latency (ahead of). Figure 12 also shows that the distribution of remote areas has a sparser layout than that of urban areas. Besides, according to Figure 11, there is not a great difference between the number of remote areas and urban areas, which indicates that the LCODC scheme does optimize the data collection coverage.

#### 5.5.2. Impacts of Data Centers

**Figure 13:**
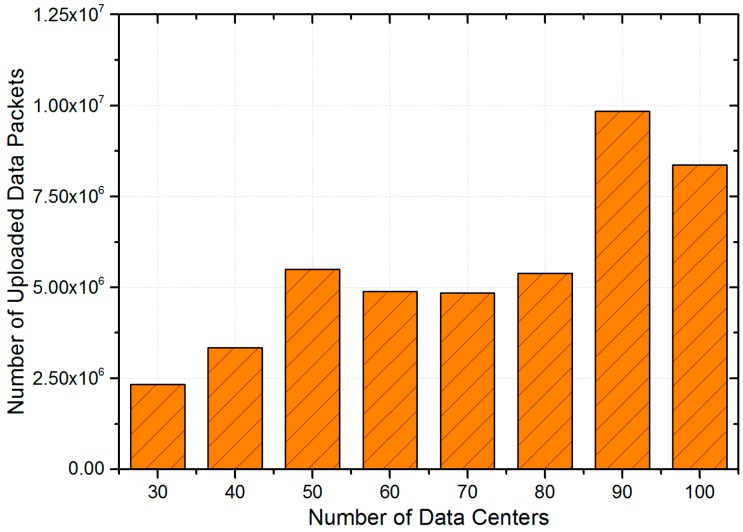
Total number of uploaded data packets with different number of data centers.

**Figure 14:**
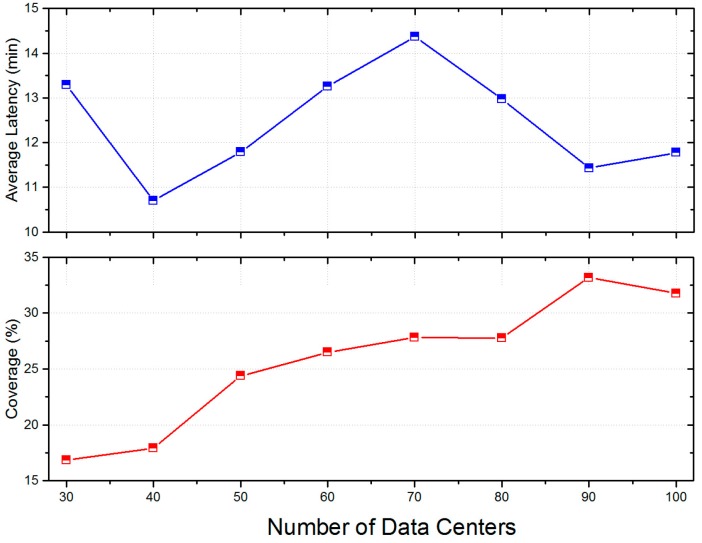
Average latency and coverage with different number of data centers.

**Figure 15:**
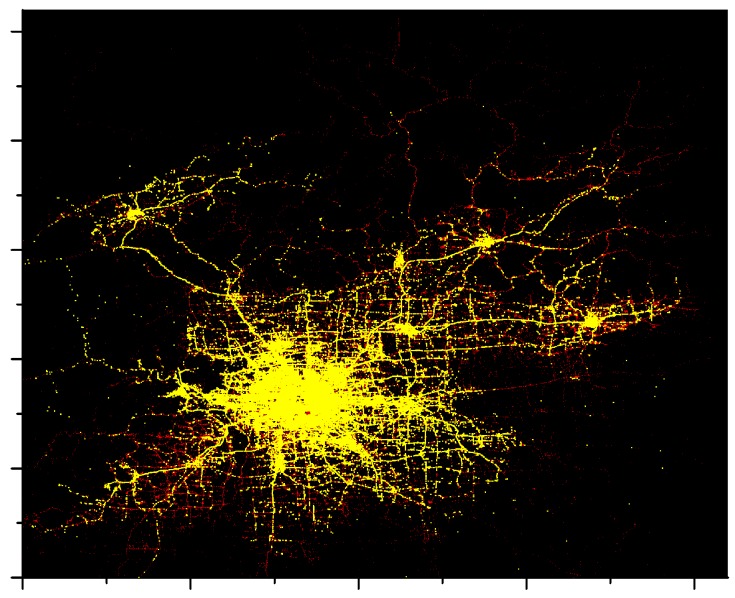
Distribution of grids covered by uploaded data packets with 100 data centers.

In the LCODC scheme, the number of data centers directly determines the hyper-parameter K in k-means and k-means++. On the other hand, Figure 8 clearly demonstrates the correlation between the locations of data centers and the final performance of the LCODC scheme. Therefore, it is necessary to analyze the impact of the number and locations of data centers. Figure 13 presents changes of the total number of uploaded data packets while Figure 14 shows changes of average latency and coverage of the LCODC scheme with different number of data centers. Besides, Figure 15 presents the distribution on grids covered by uploaded data packets when the number of data centers is set to 100. 

According to Figure 13 and Figure 14, the coverage still improves when the total number of uploaded data packets decreases (when the number of data centers ranges from 50 to 70), which cannot be clearly illustrated when taking the impacts of the number of data centers alone into consideration. Therefore, in the next part, we conduct an analysis on the locations of data centers. Besides, an increase on the number of data centers brings no improvement on the average latency according to Figure 14.

**Figure 16:**
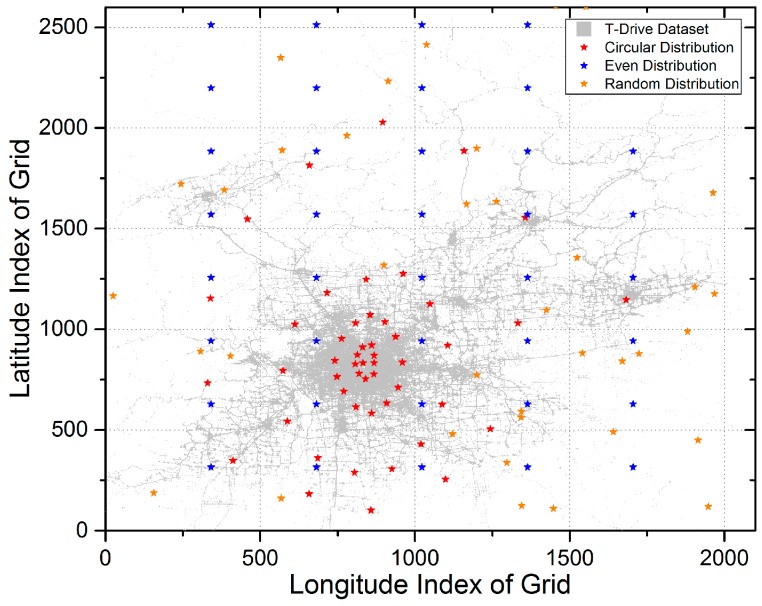
Locations of data centers with three different distributions.

To begin with, we describe three different methods on deciding the locations of data centers for comparison with the LCODC scheme: (1) locations with random distribution, which indicates that we randomly pick out 50 different grids as the locations of data centers; (2) locations with even distribution, which indicates that data centers are evenly distributed, with 10 data centers along the longitude and five data centers along latitude; (3) locations with circular distribution, which indicates that data centers present a circular shape. Besides, we modify the location of the centers of circles to conform to the real layout of Beijing. Figure 16 presents detailed information on the layout of these data centers.

As in Table 2, experimental results of data centers with three different distributions are shown in Table 4. Since a bigger proportion of data centers with circular distribution are located in urban areas compared with that in Figure 7, the number of uploaded data packets is much larger than with the original LCODC scheme. However, the original LCODC scheme greatly outperforms that scenario on coverage. Besides, due to the fact that the LCODC scheme optimizes the latency of data packets, data centers with circular distribution have no superiority in average latency.

#### 5.5.3. Impact of the Size of Internal Memory

**Figure 17:**
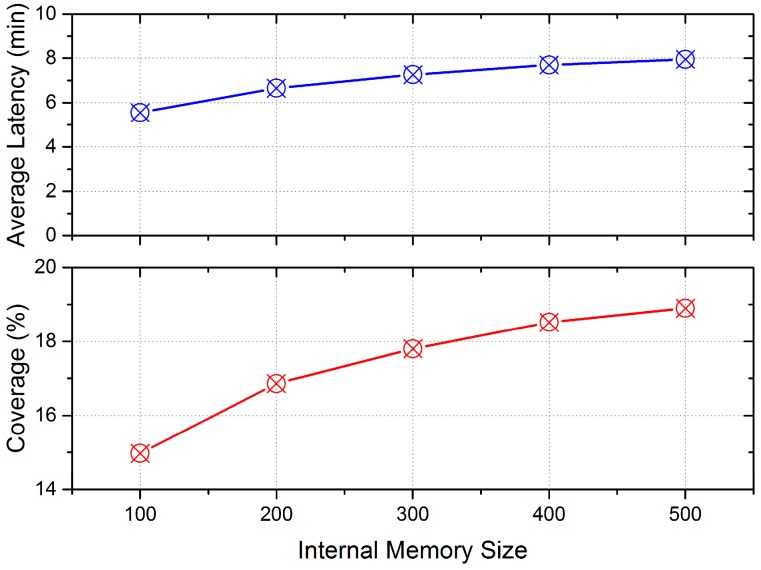
Average latency and coverage of LCODC scheme with different internal memory sizes (measured by the number of data packets).

The internal memory size directly determines the number of data packets that can be stored by mobile vehicles. Therefore, the number of packets uploaded to data centers is expected to increase with the growth on internal memory size, leading to an improvement on the coverage of the LCODC scheme. However, with more data packets to be exchanged between mobile vehicles, the time consumption will also increase. Figure 17 presents the impact of internal memory size on average latency and coverage.

#### 5.5.4. Performance at Different Times in One Day

**Figure 18:**
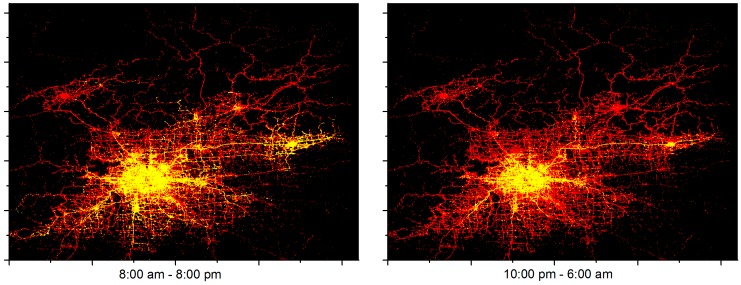
Distributions of covered grids at different times in a day.

In this part, we separately pick out two sub-datasets according to the collection time of historical trajectories in the T-Drive dataset (10:00 p.m.–6:00 a.m. and 8:00 a.m.–8:00 p.m.) to study the performance of the LCODC scheme at different times in one day. Table 5 shows the experimental results based on these two sub-datasets. Intuitively, due to a decrease on the number of active mobile vehicles, the average latency and coverage of the LCODC scheme from 8:00 a.m. to 8:00 p.m. simultaneously outperform those from 10:00 p.m. to 6:00 a.m. Besides, a great difference exists between the number of uploaded data packets at different times in a day. Figure 18 presents detailed distributions of covered grids at the two time intervals separately.

## 6. Conclusions

In this paper, we have presented a latency and coverage optimized data collection scheme for smart cities. Apart from vehicle to device transmission alone, this new scheme also includes vehicle to vehicle transmission to improve the efficiency of data collection. Besides, through mining historical trajectories of mobile vehicles to find out patterns in the smart city, this new scheme requires less public resources. We have shown through a large-scale simulation that with very limited cost, our LCODC scheme greatly reduces the latency while guaranteeing an acceptable coverage rate. In our future work, we plan to further improve the performance of the LCODC scheme through mining more kinds of historical information and developing additional mechanisms to fulfill QoS requirements.

## Figures and Tables

**Table 1:** Key parameters on the experiment.

Parameter	Value
Transmission Radius	100 m
Data Packet Size	20 bytes
Internal Memory Size	4 KB (about 200 data packets)
Time Interval of Trajectories	2 min
k in Equation (8)	1000

**Table 2:** Experimental results of LCODC scheme.

Performance Metric	k-Means	k-Means++	Mean-Shift
Number of Uploaded Data Packets	4,578,695	5,010,783	626,863
Number of Mobile Vehicles Covered by Data Packets	6954	7059	1914
Average Latency (min)	11.8564	12.9290	37.5223
Coverage (%)	22.31	24.35	9.37

**Table 3:** Comparison with other schemes.

Scheme	Average Latency (min)	Coverage (%)
LCODC (k-means)	11.8564	22.31
Scheme One	444	8.47
Scheme Two	180	27.96

**Table 4:** Experimental results of LCODC scheme with different locations of data centers.

Performance Metric	Random Distribution	Even Distribution	Circular Distribution
Number of Uploaded Data Packets	26,450	86,201	11,254,529
Average Latency (min)	72.3788	63.7886	11.6063
Coverage (%)	1.44	4.50	11.24

**Table 5:** Experimental results at different time in one day.

Performance Metric	10:00 p.m.–6:00 a.m.	8:00 a.m.–8:00 p.m.
Number of Uploaded Data Packets	1,090,251	3,593,755
Average Latency (min)	14.0951	11.3083
Coverage (%)	10.87	20.67
